# Influence of body position on diaphragmatic thickening fraction in healthy adults: advancing the standardization of diaphragm ultrasonography

**DOI:** 10.3389/fphys.2026.1765231

**Published:** 2026-02-04

**Authors:** Leonardo Arzayus-Patiño, Jorge Enrique Daza-Arana, Laura Rojas Anacona, Isabella Villota Portilla, Laura Valentina Vargas Campo, Esther Cecilia Wilches-Luna

**Affiliations:** 1 Programa de Fisioterapia, Facultad de Salud, Universidad Santiago de Cali, Cali, Colombia; 2 Doctorado en Ciencias Aplicadas, Facultad de Ciencias Básicas, Universidad Santiago de Cali, Cali, Colombia; 3 Facultad de salud, Universidad del valle, Grupo de investigación Ejercicio y Salud Cardiopulmonar GIESC, Cali, Colombia

**Keywords:** diaphragm thickness, patient positioning, physiology, ultrasonography, ultrasound

## Abstract

**Introduction:**

The diaphragm is the main respiratory muscle, and its dysfunction—particularly in hospitalized patients or those in intensive care units—is associated with difficulties in weaning from mechanical ventilation, functional deterioration, and increased healthcare costs. Although measurements such as maximal inspiratory and expiratory pressures (MIP and MEP), handgrip strength, and the Medical Research Council (MRC) scale allow for the estimation of global muscle strength, they do not directly assess diaphragmatic function. Ultrasonography has emerged as a precise and non-invasive technique for evaluating the diaphragm through parameters such as excursion, thickness, and diaphragmatic thickening fraction (DTF). However, body position during assessment introduces variability and limits protocol standardization, highlighting the need to clarify its influence in healthy individuals.

**Objective:**

To determine the influence of changes in body position on diaphragmatic thickening fraction and diaphragm thickness assessed by ultrasonography in healthy adults from the city of Cali.

**Methods:**

A cross-sectional descriptive study was conducted in 36 healthy adults (18 men and 18 women) aged 18–65 years. A high-frequency linear probe (7–18 MHz) was used to assess the right hemidiaphragm in five body positions: supine (0°) and head-of-bed elevations of 30°, 45°, 70°, and 90°. Diaphragmatic thickening fraction, thickness at end-expiration, and thickness at end-inspiration were measured. Differences between positions were analyzed using Friedman’s test with *post hoc* comparisons, and additional analyses explored associations with age and body mass index.

**Results:**

Diaphragmatic thickening fraction increased progressively with trunk inclination, reaching its highest value at 70°, followed by a significant decrease at 90° (p < 0.001). Significant differences across positions were also observed for diaphragm thickness at both end-expiration and end-inspiration (p < 0.001). Associations with age and body mass index were identified only at 90°, whereas intermediate positions showed a more stable diaphragmatic behavior. In sex-stratified analyses, significant differences were observed exclusively at 70°.

**Conclusion:**

Body position significantly influences diaphragmatic thickening fraction, with 70° producing the highest values in healthy adults. These findings highlight the need to standardize posture during diaphragm ultrasonographic assessment to optimize diagnostic precision.

## Introduction

The diaphragm is the primary respiratory muscle, and its proper functioning is essential for efficient breathing. In hospitalized patients particularly those in intensive care units respiratory muscle weakness is a frequent complication that can hinder the withdrawal of mechanical ventilation (Hermans et al., 2010). This weakness not only negatively affects patients’ quality of life but also represents an economic challenge for healthcare systems, as it significantly increases medical costs due to prolonged hospitalization and the need for additional procedures to manage associated complications (Goligher et al., 2015).

To mitigate the effects of muscle weakness, it is essential to perform appropriate assessments of both respiratory and peripheral musculature. Respiratory muscle strength is commonly evaluated using measurements such as Maximum Inspiratory Pressure (MIP) and Maximum Expiratory Pressure (MEP), while peripheral muscle strength can be assessed through tools such as handgrip strength testing and the Medical Research Council (MRC) scale (Wilches-Luna and Arzayus, 2023). These tests provide objective evaluations of functional muscle status, allowing healthcare professionals to design personalized rehabilitation plans and make informed decisions to reduce muscle weakness. Implementing these assessments in the clinical setting is fundamental for effective management, optimizing recovery, and reducing hospital length of stay.

With technological advances and the need for more precise assessments, the evaluation of respiratory musculature has become increasingly specific. Although tools such as MIP are useful, they do not isolate or directly assess the diaphragm the main muscle of respiration and require patient cooperation. In this context, ultrasonography has emerged in recent years as a valuable technique that allows direct visualization of diaphragmatic thickness and the calculation of the thickening fraction (Gil-et al., 2017). This measurement not only provides a more accurate evaluation of diaphragmatic behavior but also serves as a reliable estimate of muscle dysfunction, making it a crucial indicator for assessing one of the key components of muscle performance (Lerolle et al., 2009).

Diaphragm thickness depends on muscle mass, which correlates with force-generating capacity. Diaphragmatic thickness can be easily quantified by ultrasonography, with the right hemidiaphragm visualized through the hepatic window being more accessible than the left. To measure thickness and subsequently calculate the thickening fraction, a high-frequency linear probe (7–18 MHz) is used. This measurement allows assessment of the diaphragmatic thickening fraction (DTF), an ultrasound parameter that quantifies the change in thickness and evaluates its functional behavior (Sarwal et al., 2013).

The use of ultrasonography to assess the diaphragm has proven to be an important tool in clinical decision-making, particularly for diagnosing impairments in diaphragmatic performance. However, several studies have shown that ultrasound measurements of the diaphragm vary considerably in both healthy populations (Orde et al., 2016) and in patients (Saad et al., 2023). This variability has been attributed, in part, to the lack of standardization in body position during the ultrasound examination, as factors such as bed angle or patient posture can influence the diaphragm’s thickening fraction (Hellyer et al., 2017). This methodological inconsistency can significantly alter the values obtained and, consequently, affect clinical interpretation. Parada and colleagues, in a systematic review on diaphragm ultrasonography as a predictor of weaning, identified marked heterogeneity in the protocols used across studies, noting that many examined patients in different positions or did not specify the posture used an aspect that substantially contributes to the variability in reported thickening fraction (Parada-Gereda et al., 2023). Furthermore, the EXODUS consensus recognized that no definitive agreement was reached regarding key aspects such as the standardization of transducer and patient positioning, as multiple studies report body position as a determining factor in measurement variability (Haaksma et al., 2022). This inconsistency highlights the need for standardized evaluation protocols to ensure reliability and comparability of results, particularly in clinical contexts where these measurements are fundamental for decision-making.

Some studies indicate that body position affects ultrasound measurements (supine, seated, and standing) (Hellyer et al., 2017); however, within the range generally feasible in hospitalized patients from supine to fully seated at 90° uncertainty remains regarding which position provides the most representative and reliable values of the diaphragmatic thickening fraction.

Therefore, it is crucial to investigate how body position influences diaphragmatic ultrasound measurements, including both diaphragmatic thickness and the diaphragmatic thickening fraction (DTF). Understanding the effect of posture on these structural and functional parameters is essential to improve the accuracy and reproducibility of ultrasound assessments and to ensure that healthcare professionals can reliably interpret these measurements. Accordingly, the following research question arises: *What is the influence of changes in body position on diaphragmatic thickness and diaphragmatic thickening fraction assessed by ultrasonography in healthy adults from the city of Cali?*

## Methods

### Study design and participants

A descriptive cross-sectional study was conducted, collecting data at a single time point to describe and analyze the influence of body position on the diaphragmatic thickening fraction in a healthy population from the city of Cali. Data collection took place between January and August 2025.

The study population, recruited by convenience sampling, consisted of healthy adults residing in Cali, aged 18–65 years, distributed by sex and age ranges. The sample size was calculated based on a statistical power of 80% and a significance level of 5%, aiming to detect a minimum difference of 0.1 mm in the thickening fraction, as supported by previous studies with similar characteristics (Hellyer et al., 2017). To ensure representation across sex and age, the sample was divided into three age groups: 18–34, 35–50, and 51–65 years, with 6 men and 6 women included in each group. To account for potential losses during the study, the sample size was increased by 12.5%, reaching a total of 36 participants, all of whom completed the full set of measurements without dropouts or excluded data.

Participants were selected according to predefined inclusion criteria, which required voluntary adults of both sexes, aged 18 years or older, and considered apparently healthy. To ensure preserved respiratory function, all participants underwent a structured clinical interview aimed at identifying respiratory symptoms (including dyspnea, chronic cough, or exercise intolerance), relevant medical history, and potential respiratory risk factors such as smoking. Individuals reporting respiratory symptoms, a history of chronic pulmonary disease, or unexplained dyspnea were excluded. In addition, the Charlson Comorbidity Index was applied to rule out clinically significant comorbidities that could compromise pulmonary or diaphragmatic function.

Participants with mental, cognitive, visual, or auditory impairments that could compromise understanding of instructions or appropriate performance during the ultrasound procedure were excluded. Additional exclusion criteria included pregnancy; a history of neuromuscular disease or peripheral neuropathy; and skin irritation, sensitivity, or dermatological conditions that could interfere with the safe application of ultrasound gel. These conditions were considered potential sources of interference affecting participant safety or the validity of the measurements.

Because the right hemidiaphragm provides superior accessibility and anatomical definition through the hepatic acoustic window, only this side was evaluated to ensure optimal image quality, measurement reproducibility, and methodological consistency, in accordance with established recommendations for diaphragmatic ultrasonography.

### Data collection

Measurements were performed independently by two physiotherapists specializing in cardiopulmonary physiotherapy, each with certified training and experience in diaphragmatic ultrasound, to ensure accuracy and objectivity. The evaluators did not have access to previous measurements or each other’s records during the procedure to minimize observational bias. An additional blinding strategy was implemented: the sonographer was solely responsible for image acquisition, while a second, independent researcher, without communication with the sonographer during the procedure, recorded the diaphragmatic thickness values. The sonographer did not have access to previously recorded values or data entry at any stage of the study.

To assess the consistency of the measurements, intra- and inter-rater reliability analyses were performed before the start of the study, using the intraclass correlation coefficient (ICC), as recommended for ultrasound research. The inter-rater reliability analysis was performed using an independent group of 20 healthy subjects who were not part of the final sample. Both evaluators performed repeated measurements of the diaphragmatic thickening fraction following the same standardized protocol. Intra-rater reliability was assessed by comparing repeated measurements obtained by the same evaluator on the same subjects, while inter-rater reliability was determined by comparing measurements performed independently by both evaluators. The ICC values showed excellent reliability, with an intra-rater ICC of 0.97 (95% CI: 0.94–0.99) and an inter-rater ICC of 0.95 (95% CI: 0.91–0.98), ensuring the reproducibility and validity of the measurements.

A pilot test was also conducted with 15 subjects, different from those in the reliability test. This allowed for estimating the time required to complete each assessment, verifying the correct functioning of the equipment, evaluating the data collection format, and confirming the participants' understanding of the instructions. Following the pilot phase, the necessary adjustments were made to finalize the standard operating procedures (SOPs). Participants in the pilot study were not included in the final study sample.

Participants were recruited via social media and email, which included detailed study information and the ethics committee approval letter from the Universidad Santiago de Cali. Those who met the eligibility criteria were scheduled at the simulated hospital clinic for informed consent procedures, followed by ultrasound evaluations.

Prior to the evaluation, participants were instructed to refrain from vigorous physical activity (>5 METs) for 12 h, avoid smoking for 12 h, wear comfortable clothing, and abstain from eating for at least 3 h. Sociodemographic data, height, and weight were recorded before beginning the diaphragmatic ultrasound assessment.

The examination was performed in a quiet, well-ventilated, and temperature-controlled environment. Participants wore comfortable clothing that allowed adequate abdominal exposure and removed any metallic objects that could interfere with image acquisition. They sequentially adopted five body positions: supine at 0° and head-of-bed elevations at 30°, 45°, 70°, and 90°.

In all evaluated positions, participants remained positioned on the examination table with a backrest, maintaining the trunk aligned with the corresponding angle of inclination and both lower limbs fully extended on the table, without knee flexion or foot support on the floor. This posture was selected to standardize lower-limb positioning and to minimize additional variations in abdominal and thoracoabdominal mechanics.

All inclination angles were verified using an angle meter and confirmed by goniometry to ensure positional standardization. Diaphragmatic thickening fraction was assessed using a SonoScape E2 ultrasound system equipped with a high-frequency linear transducer.

**FIGURE 1:**
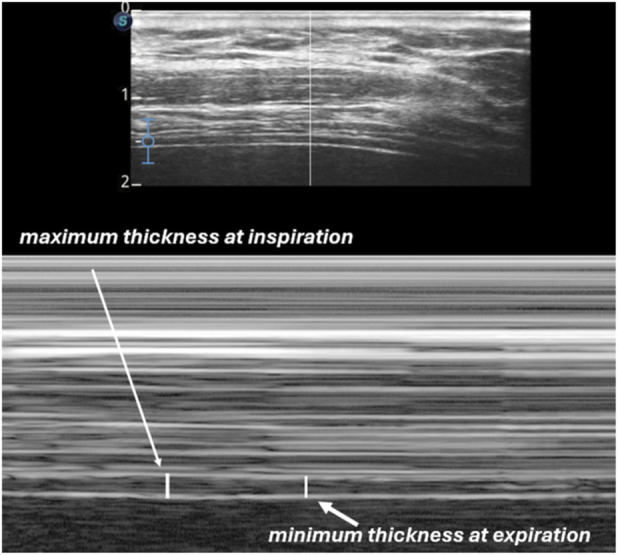
Measurement of diaphragmatic thickness using B-mode and M-mode ultrasonography. The upper schematic illustrates the anatomical landmarks and measurement points in 2D (B-mode). The lower image shows the M-mode tracing, highlighting the maximum diaphragmatic thickness during inspiration and the minimum thickness during expiration.

The measurement zone was located between the eighth and tenth intercostal spaces, corresponding to the zone of apposition. The linear transducer was positioned longitudinally along the anterior axillary line, parallel to the body’s long axis, applying minimal pressure (Scarlata et al., 2019). The diaphragm was initially identified in B-mode (2D), after which M-mode was used for measurement. Upon image freeze, the maximum inspiratory thickness and minimum expiratory thickness were recorded (Figure 1). Each position was maintained for 2 minutes prior to measurement to allow respiratory stabilization. Participants were then evaluated in all five positions, recording diaphragmatic thickness and thickening fraction. Bed inclination was verified using the integrated measurement system and cross-checked with a goniometer.

The thickening fraction (TF) was calculated using the following formula to obtain the diaphragmatic thickening fraction (Dot et al., 2017): (Maximum thickness at inspiration–Minimum thickness at expiration) × 100/Minimum thickness at expiration.

To ensure the quality of the recorded information, a computer dedicated exclusively to this purpose was used. The principal investigator performed random checks to verify consistency between the original ultrasound data, the corresponding data collection forms, and the database. Any detected errors were corrected immediately.

Additionally, a second independent investigator performed random audits to ensure accuracy before final data entry. No missing data were reported; therefore, data imputation was not required. Once the quality control process was completed, the information was entered into Excel 2020 and subsequently exported to SPSS for analysis. Statistical analyses were conducted using SPSS version 29.0 (IBM Corp., Armonk, NY, United States).

### Data analysis

A descriptive analysis was conducted to characterize the study population and evaluate the behavior of each variable. An exploratory analysis was performed using measures of central tendency, such as mean and median, as well as measures of dispersion, including standard deviation, minimum and maximum values, and interquartile range. Categorical variables are presented as frequencies and percentages. A normality test, specifically the Shapiro–Wilk test, was applied to determine the distribution of quantitative variables. To evaluate whether significant differences existed in the thickening fraction across body positions, the Friedman test was used. Post hoc comparisons were performed using the Wilcoxon signed-rank test with Bonferroni correction for multiple comparisons to identify which specific groups differed from each other. Effect size between measurements across positions was estimated using Cohen’s d for the DTF, with cutoff values interpreted as small (0.20), medium (0.50), and large (0.80) effects.

Additionally, analyses were conducted to explore the influence of anthropometric and demographic variables on the DTF. DTF values were compared across different age groups at each head-of-bed inclination evaluated (0°, 30°, 45°, 70°, and 90°). Furthermore, associations between DTF and body mass index (BMI), chest circumference, and age were examined.

As the variables did not meet the assumptions of normality, comparisons among three independent groups were performed using the nonparametric Kruskal–Wallis test. When statistically significant global differences were identified, pairwise *post hoc* comparisons were conducted using the Wilcoxon–Mann–Whitney (ranksum) test, with Bonferroni correction applied to control for type I error associated with multiple comparisons. A p value <0.05 was considered statistically significant, or the corresponding adjusted p value in *post hoc* analyses.

This study adhered to international clinical research guidelines, including the Declaration of Helsinki. Furthermore, according to Resolution 8430 of the Colombian Ministry of Health (1993), this study was classified as minimal risk research. The Ethics and Bioethics Committee of the Faculty of Health at Universidad Santiago de Cali approved the study (Record No. 20241115).

## Results

Between May and July 2025, measurements were conducted on a sample of 36 healthy individuals, of whom 50% were women. The mean age of the participants was 41.2 ± 14.2 years. The average body mass index (BMI) was 26.6 ± 3.9 kg/m^2^, with values ranging from 21.1 to 34.8 kg/m^2^. According to the International Physical Activity Questionnaire (IPAQ), all participants reported low to moderate levels of physical activity. Chest circumference showed statistically significant differences between sexes, with higher mean values observed in males (Table 1).

**TABLE 1:** Sociodemographic and anthropometric characteristics.

Variable	Fémale (n = 18)	Male (n = 18)	Total (n = 36)	*p-valué*
Age x̄ ± SD	41.4 ± 12.3	41.3 ± 14.8	41.2 *±* 14.2	0.982
Socioeconomic status				
Low (1–2)	7 (38.9%)	1 (5.6%)	8 (22.2%)	0.013
Middle (3–4)	8 (44.4%)	16 (88.8%)	24 (66.7%)	
High (5–6)	3 (16.7%)	1 (5.6%)	4 (11.1%)	
Physical activity level				
Low	17 (94.4%)	14 (77.8%)	31 (86.1%)	0.338
Moderate	1 (5.6%)	4 (22.2%)	5 (13.9%)	
BMI x̄ ± SD	26.4 ± 4.1	26.8 ± 3.8	26.6 ± 3.9	0.763
Chest circumference x̄ ± SD	79.5 ± 8.6	85.8 ± 10.3	82.6 ± 9.8	0.045

Abbreviations: x̄, mean; SD, standard deviation; BMI, body mass index.

Chi-square test used for differences in proportions; Student’s t-test used for differences in means.

When diaphragmatic thickness was analyzed, both end-expiratory thickness (Texp) and end-inspiratory thickness (Tinsp) showed variations across the evaluated body positions (0°, 30°, 45°, 70°, and 90°). End-expiratory thickness demonstrated a progressive increase with greater trunk inclination, whereas inspiratory thickness exhibited smaller variations among the different positions (Table 2). When the behavior of the DTF was analyzed according to body position, a progressive increase in median DTF values was observed as positions changed, reaching its highest value at 70°, followed by a marked decrease at 90° (Table 2). Table 2 Median DTF by body position.

**TABLE 2:** DTF and diaphragm thickness by position.

Body position	Thickening fraction, median (IQR), %	Thickness at end-expiration, median (IQR), mm	Thickness at inspiration, median (IQR), mm
0° inclination	33.0 (23.8)	1.00 (1.99)	1.35 (3.20)
30° inclination	35.0 (23.3)	1.15 (3.39)	1.70 (3.80)
45° inclination	37.0 (24.3)	1.05 (2.63)	1.60 (4.08)
70° inclination	49.5 (24.5)	1.21 (3.20)	1.60 (4.37)
90° inclination	31.5 (22.8)	1.25 (3.19)	1.60 (4.13)

When diaphragmatic thicknesses were evaluated using the Friedman test, statistically significant differences were identified across body positions for both end-expiratory thickness (Texp) and end-inspiratory thickness (Tinsp) (p < 0.001 for both). Accordingly, *post hoc* pairwise comparisons were performed using the Wilcoxon signed-rank test with Bonferroni correction. For inspiratory thickness (Tinsp), statistically significant differences were observed between the supine position (0°) and the 30°, 45°, 70°, and 90° positions, as well as between the 45° and 70° positions. Similarly, for expiratory thickness (Texp), significant differences were found between the supine position (0°) and the 30°, 45°, 70°, and 90° positions, in addition to differences between the 45° and 70° positions and between 45° and 90°.

**FIGURE 2:**
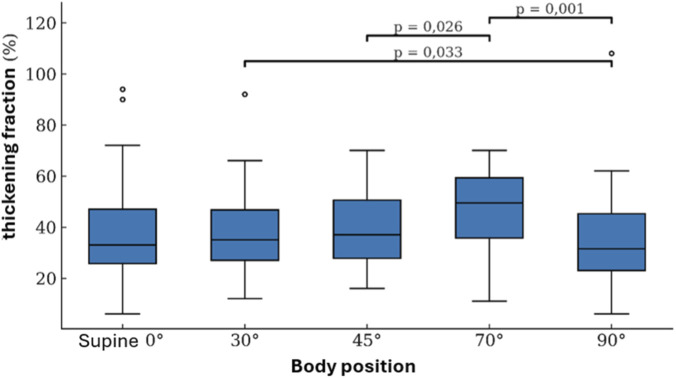
Behavior of the DTF across measurement positions.

The analysis of the behavior of the DTF showed statistically significant differences among the various body positions according to the Friedman test (p = <0.001). Post hoc comparisons, performed using the Wilcoxon signed-rank test with Bonferroni correction for multiple comparisons, revealed that the 70° position demonstrated the highest TF values, with significant differences compared to the 45° (p = 0.026) and 90° positions (p = 0.001). Additionally, a significant difference was observed between the 90° and 30° positions (p = 0.033) (Figure 2). These positional changes were associated with small effect sizes (Cohen’s d) between 30° and 90° (0.43), and between 45° and 70° (0.45), and a medium effect between 70° and 90° (0.66).

Table 3 presents the median and IQR values of the DTF by sex across the different body positions. When comparing sexes using the Mann–Whitney U test, a statistically significant difference in TF was observed only at the 70° position, with higher values in males (p = 0.009).

**TABLE 3:** Comparison of DTF by sex across different body positions.

Body position	Female Median (IQR)	Male Median (IQR)	*p-valu*e^a^
0° inclination	34.0 (32.7)	33.0 (16.0)	0,791
30° inclination	32.5 (17.7)	37.5 (17.8)	0,424
45° inclination	33.0 (25.0)	37.0 (18.5)	0,443
70° inclination	36.0 (23.2)	54.5 (10.5)	0,009
90° inclination	31.5 (25.0)	31.5 (21.5)	0,913

^a^ Test de Mann-Whitney U.

When comparing the DTF among the three age groups (18–34, 35–50, and 51–65 years), no statistically significant differences were observed at head-of-bed inclinations of 0°, 30°, 45°, and 70° (p > 0.05 for all comparisons). Across these positions, median DTF values exhibited a similar pattern among age groups, characterized by a progressive increase with changes in body position and reaching a maximum at 70°.

In contrast, at the 90° inclination, statistically significant differences were identified among age groups (p = 0.018), with progressively lower DTF values observed in the oldest group (51–65 years) compared with the younger groups.

Regarding anthropometric variables, body mass index (BMI) differed significantly among age groups (p = 0.015), with higher median values observed in the older groups. In contrast, chest circumference did not show statistically significant differences among the evaluated age groups (p = 0.540) (Table 4).

**TABLE 4:** Diaphragm thickening fraction by age groups, body mass index, and chest circumference.

Body position	Group 1 median (IQR)	Group 2 median (IQR)	Group 3 median (IQR)	*p value*
0° inclination	31.5 (16.0)	34.0 (29.5)	30.0 (31.5)	0.376
30° inclination	31.5 (15.5)	48.5 (28.0)	33.0 (16.5)	0.131
45° inclination	33.0 (21.0)	41.5 (21.5)	37.0 (25.0)	0.580
70° inclination	51.5 (25.5)	51.5 (20.0)	38.0 (30.5)	0.293
90° inclination	39.0 (24.5)	38.0 (16.0)	21.5 (12.0)	0.018

Variable	Group 1 median (IQR)	Group 2 median (IQR)	Group 3 median (IQR)	p value
Body mass index (kg/m^2^)	24.4 (3.8)	26.9 (5.1)	27.5 (4.5)	0.015
Chest circumference (cm)	79.0 (8.7)	82.0 (17.0)	82.5 (11.8)	0.540
Kruskal–Wallis test				

Age groups: Group 1 = 18–34 years; Group 2 = 35–50 years; Group 3 = 51–65 years.

**FIGURE 3:**
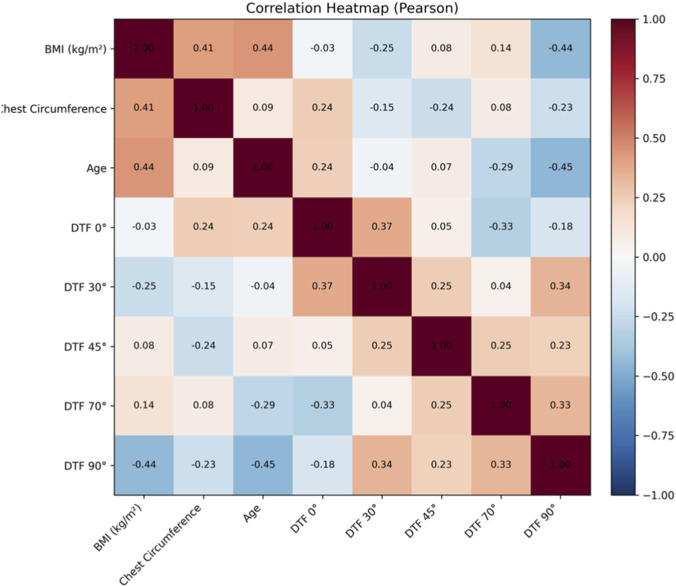
Diaphragm thickening fraction across body positions by age groups.

The Pearson correlation analysis between anthropometric variables (body mass index [BMI] and chest circumference), age, and DTF across the different body positions is presented in Figure 3.

BMI showed a significant correlation with chest circumference (r = 0.41; p = 0.013) and with age (r = 0.44; p = 0.008). Regarding DTF, no statistically significant correlations were observed between BMI and DTF at 0°, 30°, 45°, or 70° (p > 0.05 in all cases). However, at the 90° position, a moderate and statistically significant inverse correlation was identified between BMI and DTF (r = −0.44; p = 0.006).

Chest circumference did not show significant correlations with DTF at any of the evaluated positions (p > 0.05). Similarly, age was not significantly associated with DTF at 0°, 30°, 45°, or 70° (p > 0.05), whereas at the 90° position a moderate and statistically significant inverse correlation was observed (r = −0.45; p = 0.006).

The correlation coefficients and their corresponding significance values for all analyzed variables are graphically summarized in Figure 3.

## Discussion

The primary objective of this study was to determine the influence of body position on the diaphragmatic thickening fraction (TF), assessed through ultrasonography, in a healthy population from the city of Cali. The results demonstrated a significant influence, with a progressive and linear increase in TF as the body position changed, reaching its highest value at 70° of inclination.

This behavior could be explained by changes in diaphragm length and thickness within the thoracic cavity, influenced by gravitational effects on abdominal and thoracic viscera. In the supine position, the abdominal organs exert upward pressure on the diaphragm, potentially elongating it at rest and limiting its ability to thicken and generate force during inspiration. As a more upright position is adopted, this pressure decreases, allowing the diaphragm to move in a less constrained environment and partially in a direction favored by gravity, facilitating contraction. This pattern aligns with the observed progressive increase in TF, peaking at 70°.

The diaphragm is a muscle with multiple anatomical insertions and complex contractile behavior that has been described through various biomechanical theories. One such theory proposes that its contractile capacity is partly determined by external forces acting on it, particularly those generated by adjacent structures and gravity. Chen and Boriek (2022) developed a theoretical framework modeling the diaphragm as an elastic surface with anisotropic properties. This model suggests that diaphragmatic motion and thickening depend on the resulting force vector, which is influenced by deformation geometry, anisotropic material properties, and the muscle activation parameter (Chen and Boriek, 2020). Furthermore, its three-dimensional geometry, radius of curvature, extent of the zone of apposition (ZOA), force–length properties, and abdominal wall compliance may all affect this vector. Since body position modifies these forces, it becomes a determinant of diaphragmatic force generation, highlighting the importance of identifying the posture in which the diaphragm performs most efficiently (Feletti et al., 2020).

The finding that TF peaked at 70° is consistent with the theory of gravitational displacement of abdominal contents. A similar result was reported by Brown et al. (2018), who found statistically significant differences in diaphragmatic contractility—assessed via ultrasound—when comparing the supine, supported sitting, and standing positions in healthy subjects. Their study showed that in the supine position, abdominal viscera exert upward pressure on the diaphragm, limiting its ability to thicken and generate force during contraction. Although intermediate positions were not included in their study, they concluded that upright positions yield higher values, which aligns with our findings. However, the inclusion of intermediate angles in our study revealed that 70° provides a biomechanical advantage superior to both extreme positions (Brown et al., 2018).

Our study also identified a decrease in TF when comparing 90°–70°. This reduction may be explained by the interaction between posture and intra-abdominal pressure. In a fully seated 90° position with hip flexion at the same angle, although the abdomen is partially descended, its mobility may be constrained by anterior compression. This configuration can increase intra-abdominal pressure, unlike in standing, where abdominal contents are freer to descend. The resulting increase in intra-abdominal pressure at 90° may oppose the caudal displacement of the diaphragm during inspiration, restricting its thickening capacity. This finding has not been previously reported in the scientific literature based on our review, although increased intra-abdominal pressure (IAP) in this position has been documented (Brown et al., 2018).

When comparing the 90° and 0° positions in our study, TF at 90°—although lower than at 70°—was significantly higher than in the supine position. This aligns with prior studies comparing supine, sitting, and standing positions (Hellyer et al., 2017), (Brown et al., 2018). Brown et al. (2018) reported TF values of 60% in supine, 97% in sitting, and 174% in standing positions, suggesting that greater verticality favors diaphragmatic thickening during inspiration (Brown et al., 2018). Similarly, Hellyer et al. (2017) found that diaphragmatic thickness measured at end-expiratory lung volume (EELV) and at total lung capacity (TLC) was over 20% greater in sitting and standing positions compared to supine (Hellyer et al., 2017). These observations support the premise that body position significantly influences diaphragmatic mechanics and that measurements should be taken in the most biomechanically favorable postures.

Our results demonstrated statistically significant differences in TF when comparing 70° with both 45° and 90°, indicating that 70° is the optimal position for ultrasound assessment of this variable, followed by 45°. This suggests that intermediate positions favor better diaphragmatic mechanics, possibly due to a more balanced distribution of intra-abdominal pressure and greater freedom of diaphragmatic displacement. Clinically, measurement at 70° is recommended whenever feasible, as it produced the highest TF values. However, when a more stable and easily reproducible posture is required, 45° is a valid alternative. In contrast, extreme positions such as supine (0°) and full sitting (90°) are not advisable, as they showed significantly lower values than intermediate positions.

Finding significant differences in TF according to body position confirms that posture directly influences diaphragmatic activity during ultrasound assessment. This is especially relevant since there is no consensus on the most appropriate posture for measuring diaphragmatic thickness in healthy subjects. Although the EXODUS consensus (Haaksma et al., 2022) provides technical recommendations for ultrasound in critically ill patients, it gives limited attention to posture despite acknowledging that normal values vary significantly with body position. Several studies have used the supine position to evaluate diaphragmatic thickness in healthy subjects (Carillo, 2016), (Zhang et al., 2024), arguing for its reproducibility and lower variability (Vieiria, 2020). Möller et al. (2025) also consider supine to be the most pragmatic option in critically ill patients (Möller et al., 2025). Nonetheless, our results suggest that supine is not the most functionally advantageous position, as it yielded the lowest TF values compared to intermediate angles such as 70° and 45°, findings consistent with studies using semi-Fowler’s positions (30°–45°) (Bellissimo et al., 2023)– (BALDWIN et al., 2011).

Baldwin et al. (2011) reported that supine positions may alter measurement reliability due to decreased lung volume, which modifies diaphragmatic dimensions, thickening, and contractility (BALDWIN et al., 2011). In our study, transitioning from 45° to 70° resulted in a clinically relevant increase in TF. Thus, if the goal is to assess maximal functional capacity, 70° appears optimal; however, for reproducibility or longitudinal follow-up, 45° or 30° may be more appropriate. This suggests that TF assessment should prioritize intermediate angles, which may represent more physiological alternatives when patient condition permits.

When diaphragmatic thicknesses were analyzed separately, both end-expiratory thickness (Texp) and end-inspiratory thickness (Tinsp) were found to vary significantly according to body position. However, postural changes produced a more pronounced effect on expiratory thickness than on inspiratory thickness, suggesting that body position more strongly influences the basal resting state of the diaphragm than its thickness during active contraction. This finding may be explained by posture-related changes in intra-abdominal pressure and passive muscle length, which primarily affect the thickness measured at end expiration. From a clinical perspective, these results indicate that diaphragmatic thickness particularly Texp is highly sensitive to positional changes and therefore more vulnerable to the lack of postural standardization. This underscores the importance of systematically controlling and reporting body position when assessing diaphragmatic thickness by ultrasonography, as well as interpreting structural measurements (Texp and Tinsp) alongside functional parameters such as the thickening fraction to achieve a comprehensive evaluation of diaphragmatic behavior.

Analysis by sex showed no significant differences in TF between males and females across most positions except at 70°. This difference may reflect the interaction between sex-specific thoracoabdominal characteristics and the mechanical influence of posture on diaphragmatic function. Healthy male subjects are known to have greater diaphragmatic muscle thickness compared with females (Carillo, 2016), (Kharma, 2020). Men also tend to have greater visceral fat volume, which affects intra-abdominal pressure and the diaphragm–abdomen relationship (Després, 2012), (Sartorio et al., 2016). Furthermore, more upright postures facilitate abdominal content descent and reduce mechanical opposition to diaphragm contraction (Brown et al., 2018), (Boussuges et al., 2009). At 70°, these anatomical and functional differences may become more pronounced, whereas at lower or more extreme positions (0°, 30°, 45°, 90°) mechanical constraints may homogenize these differences. This is consistent with studies reporting no sex differences in diaphragmatic behavior (Hellyer et al., 2017), (Zhang et al., 2024), (Yamada et al., 2024). Therefore, TF assessment in intermediate positions such as 70° or 45° is recommended regardless of sex.

This research provides valuable clinical evidence and is, to our knowledge, the first study to systematically evaluate diaphragmatic TF across intermediate body positions. The findings help identify the posture that optimizes TF measurement, improving diagnostic precision and enabling more reliable monitoring of diaphragmatic function across clinical settings, including respiratory muscle training, physiological research, and potentially in critically ill patients.

Although the diaphragmatic thickening fraction reached its highest values at 70° in healthy subjects, these findings should be interpreted within a physiological and methodological framework and do not imply a direct clinical recommendation for patient assessment. Rather, they highlight body position as a key variable influencing diaphragmatic ultrasound measurements and underscore the need for its systematic control and reporting. Establishing a baseline physiological pattern of diaphragmatic behavior across body positions in healthy individuals represents an important initial step toward standardizing this measurement. These data may serve as a reference for future investigations in clinical settings, such as intensive care units, where optimal postures may not always be feasible and where the thickening fraction is frequently used to support clinical decisions, including assessment of weaning readiness, monitoring of ICU-acquired weakness, and evaluation of response to respiratory muscle training. Nevertheless, further studies in clinical populations are required before determining the applicability of these findings to patient evaluation.

When exploring the potential influence of anthropometric and demographic variables, the results showed that body mass index (BMI) and age were not significantly associated with the diaphragmatic thickening fraction (TF) in the supine position or at low and intermediate trunk inclinations (0°, 30°, 45°, and 70°). This finding may be partly explained by the relatively small sample size and the limited representation of individuals with a wide range of body compositions, particularly at the extremes of BMI. Therefore, future studies including populations with greater anthropometric variability are warranted to further clarify the relationship between body composition and diaphragmatic thickening.

However, at a trunk inclination of 90°, a moderate inverse correlation was observed between TF and BMI, as well as between TF and age, indicating that higher BMI and older age were associated with lower diaphragmatic thickening in the fully upright position. These associations should be interpreted with caution, as they may reflect posture-dependent mechanical constraints rather than intrinsic diaphragmatic dysfunction. Factors such as increased intra-abdominal pressure or reduced thoracoabdominal compliance in individuals with higher BMI or advanced age could contribute to these findings, particularly in more extreme postural conditions.

Consistently, the age-group analysis revealed statistically significant differences only at the 90° inclination, with lower TF values observed in the oldest age group, whereas diaphragmatic thickening remained relatively similar across age groups at low and intermediate inclinations. Taken together, these results suggest that intermediate trunk positions, particularly 70°, may allow the assessment of diaphragmatic thickening with less apparent influence from age- and BMI-related factors. This observation supports the importance of controlling and reporting body position in diaphragmatic ultrasound studies, rather than implying a direct clinical recommendation.

Strengths of this study include being the first to assess DTF via ultrasonography across multiple semi-seated and seated intermediate positions, beyond traditional comparisons between supine and standing. Measurements were conducted by trained evaluators with excellent inter-rater reliability, and standardized protocols ensured methodological consistency. The sample included adults of both sexes across a wide age range (18–65 years), enhancing external validity. Rigorous quality control strengthened internal validity.

Among the study limitations, the use of convenience sampling and the uniformly low physical activity levels of the participants may limit the generalizability of the findings. Although body mass index (BMI) was measured and analyzed, the sample included a relatively narrow range of BMI values, which may have reduced the ability to detect stronger associations between body composition and diaphragmatic mechanics. In addition, abdominal circumference was not assessed, precluding a more direct estimation of intra-abdominal pressure and its potential influence on diaphragmatic thickening. The limited availability of prior studies examining diaphragmatic thickening fraction across intermediate body positions also constrained direct comparisons.

Future studies should include a broader range of body compositions, incorporating measures such as abdominal circumference, visceral adiposity, and lean mass, as well as individuals with different physical activity levels. Additionally, validation of these findings in clinical and critically ill populations is needed to establish more precise reference values and to further clarify interindividual variability in diaphragmatic function across body positions.

## Conclusion

This study demonstrated that changes in body position significantly influence the diaphragmatic thickening fraction, with the highest DTF observed at 70°. These findings support the importance of considering posture as a determining variable in the functional assessment of the diaphragm using ultrasonography.

## Data Availability

The datasets presented in this article are not readily available because sin restricciones. Requests to access the datasets should be directed to leonardoarzayus00@usc.edu.co.
